# Cancer du sein traité par chimiothérapie première: facteurs prédictifs du traitement radical (étude rétrospective à propos de 72 cas)

**DOI:** 10.11604/pamj.2020.36.174.24036

**Published:** 2020-07-10

**Authors:** Ahmed Hajji, Dhekra Toumi, Amira Daldoul, Manel Njima, Houda El Mhabrech, Raja Faleh

**Affiliations:** 1 Department of Gynecology and Obstetrics, Fattouma Bourguiba University Hospital, Monastir, Tunisia,; 2 Department of oncology, Fattouma Bourguiba University-Hospital, Monastir, Tunisia,; 3 Department of Pathology, Fattouma Bourguiba University-Hospital, Monastir, Tunisia,; 4 Department of Radiology, Hadj Ali Soua Hospital, Ksar Hellal, Monastir, Tunisia

**Keywords:** Cancer du sein, chimiothérapie, facteurs prédictifs, mastectomie, Breast cancer, chemotherapy, predictive factors, mastectomy

## Abstract

La chimiothérapie néoadjuvante est devenue le traitement de première intention des cancers du sein localement avancés. Elle permet d’une part, une augmentation des chances de conservation mammaire sans pour autant une majoration du risque de récidive, et d’autre part un contrôle locorégional des formes inopérables d’emblée. Cependant, certains facteurs cliniques, radiologiques et histologiques sont associés à une augmentation du risque de mastectomie, tels que la présence de microcalcifications, la multifocalité, le grade SBR1 et 2, les stades cT3 et cT4 et la surexpression d’HER2. A partir de ce travail, nous avons cherché à déterminer les facteurs prédictifs de mastectomie après chimiothérapie néoadjuvante (CNA), si la mastectomie était justifiée ou non histologiquement et quels étaient les facteurs prédictifs de mastectomie non justifiée. Nous avons réalisé une étude rétrospective colligeant 72 patientes atteintes de cancer du sein et traité par CNA aux départements de gynécologie et d’oncologie médicale au CHU Fattouma Bourguiba de Monastir-Tunisie. Le taux du traitement conservateur était de 18,1%, il passe à 63,15% pour les tumeurs stade T2. La mastectomie n´était pas justifiée par l’histologie définitive dans 26,3% des cas. Les facteurs prédictifs de mastectomie non justifiée retrouvés dans notre étude étaient le statut RH négatif et le stade cT2. La réalisation de ce travail a conduit à une réflexion sur nos pratiques et leurs modifications. Le traitement chirurgical conservateur devra être considéré comme un standard thérapeutique et être systématiquement proposé à toutes les patientes traitées par CNA pour un cancer du sein y compris en cas de multifocalité, une taille clinique importante, des microcalcifications étendues, dans le but de diminuer de façon conséquente le nombre de mastectomies non justifiées.

## Abstract (en)

Neoadjuvant chemotherapy has become the first-line treatment for locally advanced breast cancers. On the one hand, it allows for increasing options of breast conservation without an increased risk of recurrence, on the other hand it allows for locoregional control of patients with inoperable cancer. However, some clinical, radiological and histological factors are associated with an increased risk of mastectomy, such as microcalcifications, multifocality, SBR1 and 2 grade, cT3 and cT4 stages and overexpressed HER2. The purpose of this study was to determine the predictive factors for mastectomy after neoadjuvant chemotherapy (NAC), whether mastectomy was justified or not histologically and what were the predictors for unjustified mastectomy. We conducted a retrospective study of 72 patients with breast cancer treated by neoadjuvant chemotherapy in the Departments of Gynecology and Medical Oncology at the Fattouma Bourguiba Hospital in Monastir, Tunisia. The rate of conservative treatment was 18.1%; 63.15% for stage T2 tumors. Mastectomy was not justified by definitive histologic diagnosis in 26.3% of cases. In our study, unjustified mastectomy predictors were negative RH status and CT2 stage. This study led to reflection on our practice and its modifications. Conservative surgery should be considered as standard therapy and should be routinely suggested to all patients treated with neoadjuvant chemotherapy for breast cancer, including the cases with multifocality, large clinical tumor size, extensive microcalcifications, in order to significantly reduce the number of unjustified mastectomies.

## Introduction

La prise en charge des cancers du sein localement avancés (CSLA) est pluridisciplinaire. L´objectif principal reste l´amélioration du pronostic et donc de la survie moyennant une limitation de la morbidité des traitements et l´amélioration de la qualité de vie des patientes. La chimiothérapie néoadjuvante (CNA) est devenue le traitement de référence des cancers du sein localement avancés. Elle permet de réduire la taille tumorale et offre une meilleure chance pour un traitement conservateur chez les patientes initialement candidates à une mastectomie ou inopérables [1, 2]. Certes ce traitement conservateur respecte mieux la qualité de vie des patientes, cependant, il ne doit pas se faire à travers un risque de récidive locale. A travers ce travail, nous avons essayé de déterminer les facteurs prédictifs de mastectomie après chimiothérapie néoadjuvante, ainsi que des mastectomies non justifiées histologiquement.

## Méthodes

Il s´agit d´une étude rétrospective mono centrique, menée au Département de Gynécologie et d´Oncologie à l´hôpital Fattouma Bourguiba de Monastir en Tunisie sur une période de 4 ans colligeant 72 patientes. Les critères d´inclusion regroupaient l´ensemble des patientes de sexe féminin atteintes d´un CSLA non métastatique et traitées par CNA. Dans tous les cas, la tumeur était considérée trop volumineuse pour pouvoir bénéficier d´un traitement chirurgical conservateur d´emblée. Nous avons exclu les patientes ayant des cancers du sein bilatéraux et celles pour lesquelles la mastectomie a été réalisée par choix alors qu´un traitement conservateur aurait été possible. Le diagnostic était établi par une microbiopsie écho guidée. La possibilité ou non d´un traitement conservateur était envisagée avant le début de la CNA. Un tatouage percutané a été réalisé en début du traitement au centre de la tumeur afin de pouvoir la localiser après la fin de la chimiothérapie. Toutes les patientes recevaient une chimiothérapie administrée par voie intraveineuse toutes les 3 semaines après avoir éliminé les éventuelles contre-indications aux différentes molécules. Le protocole utilisé associait 3 à 4 cycles FEC100 (5-Fuoro-Uracile, Epirubicine et Cyclophosphamide) suivi de 3 à 4 cycles Taxotère (TXT). Exceptionnellement, 6 FEC100 ou 3 FEC100, 4 TXT en raison d´intolérance ou de certaines contre-indications. La réponse à la chimiothérapie était évaluée cliniquement selon les critères définis par l´UICC, la réponse tumorale était considérée complète lorsqu´il ne persiste aucune lésion cible et partielle lorsque la réduction de la lésion cible était d´au moins 30%. La progression était définie par une augmentation de plus de 20% selon les derniers critères RECIST version 1.1 [3, 4].

Le traitement chirurgical était réalisé 4 à 6 semaines à distance de la dernière cure et après discussion du dossier en réunion de concertation pluridisciplinaire. Nous avons considéré 3 groupes en fonction du type de chirurgie réalisée: a) dans le groupe M1: un traitement chirurgical radical était décidé sans prendre en considération le type de la réponse à la CNA en cas de multifocalité, multicentricité, foyers de microcalcifications étendus, cancer du sein inflammatoire initialement (N = 36). Le traitement chirurgical était adapté à la réponse tumorale pour le reste de la population. Ainsi, nous avons distingué:

a) **le groupe M2 (mastectomie secondaire):**la mastectomie était réalisée selon la technique de Madden conservant le muscle pectoral en cas de lésion progressive sous chimiothérapie, une taille tumorale dépassant 5 cm, un rapport taille tumeur/taille sein élevé avec un résultat esthétique jugé médiocre (N = 10).

b) **le groupe C:**traitement conservateur. Les patientes ne présentant aucun critère indiquant la mastectomie avaient bénéficié d´une tumorectomie et étaient informées du risque de ré intervention en cas de limites non saine (N=26). Dans tous les cas, un curage axillaire standard (niveau 1 et 2 de Berg) était associé. Une irradiation de la glande mammaire était indiquée systématiquement après traitement conservateur. L´irradiation de la paroi thoracique après mastectomie était indiquée en cas de facteurs de mauvais pronostic. L´irradiation des aires sus et sous-claviculaires était préconisée en cas d´envahissement axillaire. L´hormonothérapie était indiquée pour les tumeurs RH+. Les patientes non ménopausées recevaient du tamoxifène et les patientes ménopausées recevaient un inhibiteur de l´aromatase seul ou après tamoxifène. Nous avons ensuite divisé la population en sous-groupes selon que le type de chirurgie était adéquat ou non à travers les résultats histologiques définitifs: a) **groupe M1**: Les mastectomies justifiées concernaient les mastectomies ypT2 à ypT4. Les mastectomies non justifiées concernaient les mastectomies ypT0 ou ypT1, quel que soit le statut ganglionnaire et qui auraient pu bénéficier d´un traitement conservateur; b) **groupe C**: la chirurgie conservatrice justifiée concernait les tumorectomies ayant permis l´exérèse tumorale en marges saines. Une chirurgie conservatrice est non justifiée lorsqu´une reprise chirurgicale radicale ou conservatrice était nécessaire. C) **groupe M2**: les mastectomies justifiées concernaient les mastectomies ypT2 à ypT4 et les mastectomies non justifiées concernaient les mastectomies ypT0 ou ypT1. Les comparaisons des groupes C versus M2 et mastectomie secondaire non justifiée versus mastectomie justifiée étaient réalisées pour les variables quantitatives par le test non paramétrique de Wilcoxon. Pour les variables qualitatives, le test de Chi-2 ou le test de Fisher étaient utilisés lorsque les effectifs le justifiaient. Lorsque les effectifs de variables catégorielles à plus de 2 modalités ne permettaient pas l´utilisation du Chi-2, des regroupements étaient fait pour obtenir un tableau à 4 cases adaptées au test de Fisher. Ces regroupements étaient décidés sur des arguments cliniques. Un p < 0,05 en test bilatéral était considéré comme significatif.

## Résultats

L´âge moyen de notre population était de 48,68 ans avec des extrêmes allant de 29 à 76 ans. 45,8% des patientes étaient ménopausées. Le délai moyen de consultation était de 4,97 mois. Le motif de consultation le plus fréquent était la découverte d´un nodule du sein (75%) mais les circonstances étaient souvent intriquées. Dans 38,9% des cas la tumeur siégeait au niveau du quadrant supéro-externe. La taille clinique moyenne était de 5,7 cm. Selon la classification TNM, La tumeur était classé T2 et T3 dans 50% des cas, 36 patientes avaient une tumeur T4 dont 22 patientes T4d. un envahissement ganglionnaire était objectivé dans 38,9% des cas dont 6,9% N2. Il s´agissait d´un carcinome infiltrant de type non spécifique dans 98,6% des cas, de grade SBRIII dans 31,9% des cas. Les RH étaient positifs dans 68% des cas et le HER2 était surexprimé chez 29 patientes (40,3%). Neuf patientes (12,5%) présentaient une tumeur triple négative. Une chimiothérapie séquentielle par FEC 100 suivi de Taxanes était administrée chez 95% des patientes avec une moyenne de 6,43 cures. L´examen avait objectivé une réponse clinique chez 91,6% des patientes (36,1% avaient une réponse complète (cCR) et 55,5% une réponse partielle), une maladie stable dans 7% des cas et une progression dans 1,4% des cas. Une réponse pathologique complète (pCR) était observée chez 19,4% de la population. Après la chimiothérapie 40,3% des patientes n´avaient pas un envahissement ganglionnaire. Un traitement conservateur était réalisé chez 13 patientes (18,1%). Le taux de conservation mammaire pour les tumeurs T2 était de 63,15% et de 36,1% pour T2-T3. Une mastectomie était pratiquée chez 59 patientes (d´emblée chez 46 patientes et de rattrapage chez 13 patientes). Une reprise chirurgicale pour des marges tumorales était réalisée chez 3 patientes, il s´agissait d´une mastectomie 2 fois sur 3. La taille moyenne du résidu tumoral infiltrant était de 1,73 cm (0 à 12,5 cm). Le taux de réponse pathologique complète mammaire (stade TA de Sataloff) était de 19,4%. La réponse ganglionnaire était complète (NA selon Sataloff) dans 9,7% des cas. Parmi les 72 patientes de notre population, 46 ont eu une mastectomie (36 étaient prévues avant la CNA et incluse dans le groupe M1 et 10 en réponse à la chimiothérapie et classée dans le groupe M2) et 26 patientes ont bénéficié d´une tumorectomie décidée après CNA (groupe C). Trois patientes du groupe M1 et 2 patientes du groupe M2 avaient une tumeur multifocale. Pour les stades T4d, La CNA a permis un contrôle de la maladie et la réalisation d´une mastectomie dans tous les cas. Le taux de traitement conservateur décidé après CNA était de 72,2% (N = 26). Dans 50% des cas, c´est la taille tumorale clinique en fin du traitement qui indiquait une mastectomie. La taille tumorale classiquement admise pour permettre un traitement conservateur est de 30 mm et peut être adaptée à la taille du sein. Dans notre série, parmi les 10 indications de mastectomie sur taille tumorale, 8 avaient une taille > 3 cm.

En analyse univariée (C vs M1), les variables cliniques prédictives d´un traitement chirurgical radical étaient la taille tumorale initiale > 50 mm, les stades cT3 et cT4 et La stabilité ou la progression clinique de la lésion sous CNA (p < 0,001). Il n´y avait pas de différences significatives en terme d´âge ni en terme de statut ménopausique entre les 2 groupes (respectivement p = 0,94 et p = 0,98). La multifocalité ne présentait pas un facteur prédictif de traitement radical (p = 0,07). Le statut ganglionnaire n´était pas également un facteur prédictif de mastectomie (p = 0,67). En analyse univariée, les variables histologiques (sur biopsie initiale) prédictives d´un traitement radical étaient la présence de carcinome canalaire in situ (CCIS) quel que soit le grade (p < 0,01), le grade SBR1 et SBR2 (p = 0,003), la surexpression HER2 (p < 0,001) et les tumeurs de phénotype RH+/ HER2+ (p < 0,001). Parmi les 36 mastectomies du groupe M1, 4 étaient ypT0 et 12 étaient ypT1, soit 44,4% (N = 16) des mastectomies pouvant être considérées comme non justifiées et qui auraient pu bénéficier d´un traitement conservateur. Les facteurs prédictifs de mastectomies non justifiées dans le groupe A étaient la présence de CCIS associé (p = 0,02) et l´absence de RH (p = 0,05). Dans le groupe M2, 30% (N = 10) des mastectomies étaient considérées non justifiées. Les facteurs prédictifs de mastectomie non justifiée dans ce groupe étaient le stade cT2 (p=0,08) et les RH négatifs (p=0,03). En analyse multivariée, les facteurs indépendants prédictifs d´une mastectomie non justifiée dans le groupe M2 étaient le statut RH négatif (OR 3 ic95% [0,99-9,05]) et le stade cT2 (OR 4,2 [1,37-12,89]). Dans toute la population, environ 26,4% des patientes avaient rechuté à un moment donné de leurs suivis. Le délai moyen de récidive était égal à 14,12 mois avec des extrêmes allant de 4 à 49 mois et une médiane de 10,5. Le siège de rechute était dans 73,6% des cas, uniquement à distance de la région mammaire. Lors de la mise à jour des dossiers, 77,7% des patientes étaient vivantes en rémission complète de leur maladie. Le taux de décès était de 11,1% dans l´ensemble de la population, dont les deux tiers étaient des cancers stade T4. Dans notre série, nous n´avons pas retrouvé de différence significative en termes de survie ou de récidive, selon que la chirurgie était radicale ou conservatrice.

## Discussion

Notre étude a porté sur 72 patientes traitées pour cancer du sein stade T2 à T4 non métastatique et dont le but était d´analyser les facteurs prédictifs de traitement chirurgical radical après chimiothérapie néoadjuvante. Dans une étude menée entre 1987 et 2001 sur 594 patientes ayant un cancer du sein stade T2-T3 et éligible à un traitement conservateur [5], Rouzier a conclu en analyse multivariée aux facteurs prédictifs de traitement radical suivants : une taille tumorale initiale supérieure à 5cm, un bas grade histologique et une multicentricité, résultat que nous partageons. Dans notre population d´étude nous avons 26,4% de T2 et 23,6% de T3, pour Rouzier 50,2% et 25,1%. Rouzier avait exclu les T4 de son étude et les modalités de la CNA étaient différentes. L´un des points forts de notre analyse est l´homogénéité des protocoles de la CNA. Malgré l´adjonction des thérapies ciblées et les progrès de l´oncologie et la radiothérapie, le taux des traitements conservateurs augmente peu et s´élève dans notre série à 63,15% pour les tumeurs T2, résultat proche de celui de Mauriac en 1991 (63%) [6]. Les taux rapportés dans la littérature s´échelonnent de 35 à 90% [7] et sont corrélés essentiellement au volume tumoral initial. Pour Mauriac, une taille tumorale clinique résiduelle supérieure à 2 cm indiquait une mastectomie, chose qui ne semble plus d´actualité [6]. Fisher considérait que l´importante taille tumorale ne constituait pas elle-même un critère d´exclusion pour un traitement conservateur sauf si celui-ci était à l´origine d´un mauvais résultat esthétique en raison de la petite taille du sein [8]. Dans notre série, des lésions de CCI de grade 1 ou 2 sur la biopsie étaient associées à un traitement radical plus fréquent. Ce résultat, retrouvé par Rouzier en analyse multivariée, s´explique probablement par la faible chimiosensibilité de ces lésions [5]. Nous avons conclu que la surexpression HER2 était prédictive de mastectomie. Ce résultat est partagé par Von Minckwitz et Spanheimer qui constataient que le statut HER2+ et le phénotype RH+/HER+ étaient prédictifs de traitement chirurgical radical [9, 10]. La présence de microcalcifications à la mammographie initiale ainsi que la multifocalité étaient également des facteurs prédictifs de mastectomie dans notre série. Rouzier rapportait également des résultats similaires dans son étude [5].

Les patientes qui présentaient une lésion multifocale dans notre série étaient justiciable d´un traitement radical quel que soit la réponse à la chimiothérapie. En 2006, Oh a mené la première étude évaluant la survie des patientes présentant un cancer multifocal et/ou multicentrique et traitées par CNA et chirurgie conservatrice [11]. Il a conclu que ces patientes ne présentent pas un risque accru de récidive locorégionale par rapport aux patientes présentant une lésion unique (5% versus 7%). Gentilini a conclu également, à partir d´une série de 476 patientes dont 421 présentaient une tumeur multifocale et 55 une tumeur multicentrique traitées par chirurgie conservatrice et radiothérapie, qu´en l´absence de surexpression de HER2 ou de négativité des récepteurs hormonaux le traitement conservateur n´était pas associé à une augmentation du risque de récidive locorégionale [12]. Selon lui, il existait une place pour le traitement conservateur dans ces cancers à condition que les résultats esthétiques le permettent et qu´une reprise chirurgicale soit proposée en cas de marges tumorales. Les facteurs prédictifs de mastectomie non justifiée retrouvés dans le groupe M2 de notre étude étaient le statut RH négatif et le stade cT2. Les tumeurs de grade histologique élevé sont plus souvent RH négatif et plus chimiosensibles [13]. Par ailleurs, la taille tumorale initiale inférieure à 5 cm doit nous faire reconsidérer la place de la chirurgie conservatrice dans traitement chirurgical du cancer du sein. L´étude rétrospective d´El-Didi a été conduite dans le but d´évaluer la réponse à la CNA et l´efficacité de l´examen clinique et de la mammographie préopératoire dans la détection de ces changements. Les patientes recevaient une CNA composée de 3 cycles de FEC puis une mastectomie. L´auteur s´est intéressé à la pièce anatomopathologique de mastectomie et dans 70,4% des cas une réponse partielle ou complète était observée. Cependant, seules 26,7% de ces patientes étaient candidates à un traitement conservateur. Il conclut que l´examen clinique et la mammographie n´étaient pas adaptés pour détecter les patientes éligibles à un traitement conservateur [14]. Les taux de tumorectomies avec reprise pour mastectomies étaient élevés dans notre série (50%) par rapport à la littérature (8,9% pour Coopy [15], 7% pour l´institut Curie) alors que le taux des reprises par tumorectomie est faible par rapport à la littérature (3,8% contre 23% pour Coopy [15] et 13,5% pour Rouzier [16]). En augmentant le taux de traitement conservateur, ces chiffres risquent incontestablement d´augmenter mais l´idée d´un traitement conservateur d´épreuve systématique avec mastectomie de rattrapage nous semble plus acceptable que le risque de mastectomie non justifiée en raison du caractère mutilant de la mastectomie. Dans notre série, nous n´avons pas retrouvée de différence significative en termes de survie ou de récidive selon que la chirurgie était conservatrice ou radicale.

## Conclusion

La réalisation de ce travail a conduit à une réflexion sur nos pratiques et leurs modifications. Le traitement chirurgical conservateur devra être considéré comme un standard thérapeutique et être systématiquement proposé à toute les patientes traitées par CNA pour un cancer du sein y compris en cas de multifocalité, une taille clinique importante, des microcalcifications étendues, dans le but de diminuer de façon conséquente le nombre de mastectomies non justifiées. Seules les patientes présentant une contre-indication à la radiothérapie, une mutation génétique prédisposant au cancer du sein, une volonté de mastectomie, seront éligibles à un traitement radical. La patiente sera évidemment informée du risque de ré intervention pour mastectomie de rattrapage en cas de marges positives. Afin de diminuer le risque de récidive locale, cette démarche ne pourra être effectuée que dans des conditions strictes de réalisation moyennant la réalisation d´un bilan sénologique exhaustif avec l´aide de l´IRM, des biopsies ciblées de tous les foyers suspects, un repérage de la lésion en début du traitement (tatouage, clip), l´analyse très protocolaire et très attentive des marges chirurgicales, l´irradiation systématique du sein associée à une sur impression du lit tumoral et l´utilisation de thérapeutiques adjuvantes.

### Etat des connaissances sur le sujet

- La présence de microcalcifications, la multifocalité, le grade SBR1 et 2, les stades cT3 et cT4 et la surexpression d’HER2 sont des facteurs associés significativement à un taux de mastectomie élevé après chimiothérapie première.

### Contribution de notre étude à la connaissance

- Dans notre étude, les variables prédictives d’un traitement radical après CNA étaient la taille tumorale clinique initiale > 50 mm, les stades cT3 et cT4, la stabilité ou la progression clinique de la lésion sous CNA, la présence de carcinome canalaire in situ quel que soit le grade, le grade SBR1 et SBR2, la surexpression HER2 et les tumeurs de phénotype RH+/ HER2+;
- La multifocalité et le statut ganglionnaire initial ne présentaient pas des facteurs prédictifs de mastectomie;
- Les facteurs prédictifs d’une mastectomie non justifiée étaient le statut RH négatif et le stade cT2.
